# A nationwide population-based study on the clinical and economic burden of anastomotic leakage in colorectal surgery

**DOI:** 10.1007/s00423-023-02809-4

**Published:** 2023-01-23

**Authors:** Marie-Christin Weber, Maximilian Berlet, Christian Stoess, Stefan Reischl, Dirk Wilhelm, Helmut Friess, Philipp-Alexander Neumann

**Affiliations:** 1 Department of Surgery, Technical University of Munich, TUM School of Medicine, Klinikum rechts der Isar, Ismaninger Str. 22, 81675 Munich, Germany; 2 Department of Diagnostic and Interventional Radiology, Technical University of Munich, TUM School of Medicine, Klinikum rechts der Isar, Munich, Germany

**Keywords:** Anastomotic leakage, Colorectal surgery, Postoperative complications

## Abstract

**Aim:**

Anastomotic leakage (AL) is one of the most dreaded complications in colorectal surgery. In 2013, the International Classification of Diseases code K91.83 for AL was introduced in Germany, allowing nationwide analysis of AL rates and associated parameters. The aim of this population-based study was to investigate the current incidence, risk factors, mortality, clinical management, and associated costs of AL in colorectal surgery.

**Methods:**

A data query was performed based on diagnosis-related group data of all hospital cases of inpatients undergoing colon or sphincter-preserving rectal resections between 2013 and 2018 in Germany.

**Results:**

A total number of 690,690 inpatient cases were included in this study. AL rates were 6.7% for colon resections and 9.2% for rectal resections in 2018. Regarding the treatment of AL, the application of endoluminal vacuum therapy increased during the studied period, while rates of relaparotomy, abdominal vacuum therapy, and terminal enterostomy remained stable. AL was associated with significantly increased in-house mortality (7.11% vs. 20.11% for colon resections and 3.52% vs. 11.33% for rectal resections in 2018) and higher socioeconomic costs (mean hospital reimbursement volume per case: 14,877€ (no AL) vs. 37,521€ (AL) for colon resections and 14,602€ (no AL) vs. 30,606€ (AL) for rectal resections in 2018).

**Conclusions:**

During the studied time period, AL rates did not decrease, and associated mortality remained at a high level. Our study provides updated population-based data on the clinical and economic burden of AL in Germany. Focused research in the field of AL is still urgently necessary to develop targeted strategies to prevent AL, improve patient care, and decrease socioeconomic costs.

**Supplementary Information:**

The online version contains supplementary material available at 10.1007/s00423-023-02809-4.

## Introduction

A common yet dreaded postoperative complication in colorectal surgery is anastomotic leakage (AL), which is associated with longer hospitalization, a higher rate of reoperation, and higher overall morbidity and mortality [1, 2]. AL not only leads to a high clinical burden for the patients affected but causes significantly higher costs for hospitals and national health care systems [3]. Thus, research in the field of AL has increased over recent years with a number of records in the *PubMed* database for “anastomotic leakage” of 423 in the year 2010 and 1097 in 2020. Preoperative, tumor-associated, intraoperative, and other risk factors for AL have been identified so far [4, 5]. Research in the field thus focuses on identifying biomarkers for AL as well as finding optimal surgical techniques, biomaterials, and targeted drugs to reduce the risk of AL after gastrointestinal surgery; however, no treatment option except for diverting enterostomy exists so far to reliably prevent AL [6]. AL rates after lower gastrointestinal surgery are reported in the literature to occur in 1–19% of operations; however, reported leakage rates vary largely across studies [4, 7–9].

In 2013, the ICD (International Statistical Classification of Diseases and Related Health Problems) code K91.83 for postoperative gastrointestinal AL has been introduced to the German diagnosis-related group (DRG) system. DRG statistics data from all inpatients in German acute care hospitals are collected by the German Federal Statistical Office (DESTATIS). Microdata of the DRG statistics can be retrieved by researchers through the Research Data Centers associated with DESTATIS. These prerequisites make it possible to perform a retrospective population-based study analyzing AL rates and the resulting clinical and economic burden.

The aim of this study was to delineate current trends of AL rates in colorectal surgery in Germany by examining all inpatient cases from 2013 to 2018 based on DRG data sets. Furthermore, outcomes of patient care were assessed by studying therapeutic modalities for the clinical management of AL, mortality, hospital length of stay, and socioeconomic costs.

## Methods

### Data query and inclusion criteria

A data query through the Federal Statistical Office (DESTATIS) was performed for all inpatients undergoing colon resections (OPS 5–455) and sphincter-preserving rectal resections (OPS 5–484) from 2013 to 2018 in German acute care hospitals. Parameters retrieved were patient age and sex, main diagnosis, secondary diagnoses, postoperative complications and postoperative AL, morbidity scores, in-house mortality, therapeutic management of AL, length of hospital stay, and hospital reimbursement volume (Table S1). The Strausberg Comorbidity Score and weighed Elixhauser Score were used for the comparison of general comorbidity between patients with and without AL [10–12]. The code for the data query was written in SAS programming language according to the DESTATIS requirements. Data were retrieved through remote-controlled data processing and provided as raw data by DESTATIS [13]. The detailed methods and underlying regulations for reporting of inpatient cases in German hospitals have been previously described in detail [14–17]. In summary, all acute care hospitals in Germany are required by law to document and report every inpatient case with all relevant procedures and diagnoses, mainly for financial hospital reimbursement. The data are monitored for correctness by the medical service of the health insurance funds and stored by DESTATIS. The following data items per in-house hospital case are included in the DESTATIS database and can be queried for research purposes: main diagnosis (ICD), secondary diagnoses (ICD), procedures (OPS), age, year of birth, reason and type of admission, reason and type of discharge including in-hospital death, length of hospital stay, specialist department, Case Mix, Case Mix hospital reimbursement volume in EURO, hospital location (federal state, district, municipality, postal code), and patient residence (federal state, district, municipality, postal code). No temporal information regarding the sequence of procedures or diagnosis within one hospital case and no patient follow-up data can be retrieved from the database. Raw data from data queries are provided as pooled data (number of cases for defined combinations of ICD and OPS codes). For secondary data analysis used in this study, no ethics committee statement is required [18]. For data protection purposes, case numbers ≤ 2 are blinded by DESTATIS and not available to the authors.

### Statistics

GraphPad Prism Version 9.1.2 (GraphPad Software, CA, USA) was used for statistical testing and data visualization. Fisher’s exact test, chi-square test, chi-square test for trend, and odds ratio were calculated. *T*- and Wilcoxon-signed rank tests were performed within the query code. Continuous parameters and variables are presented as mean with single standard deviation. Data were analyzed descriptively for each year and presented either as absolute numbers or relative rates. This study was conducted and reported using the STROBE Statement checklist [19].

## Results

### Baseline characteristics

**Fig. 1:**
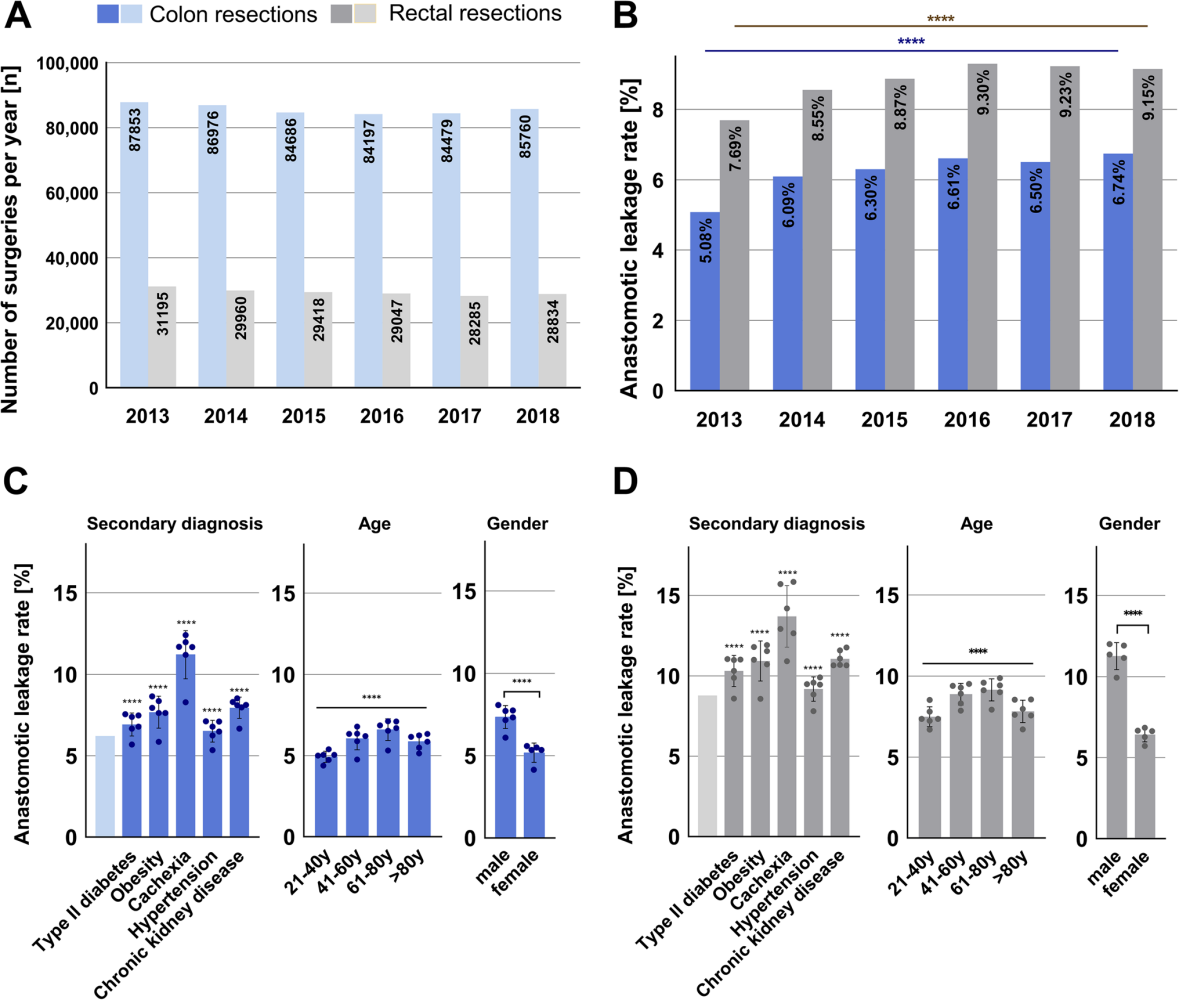
Development of surgery numbers from 2013 to 2018 for colon resection and sphincter-preserving rectal resection, anastomotic leakage rates, and risk factors. (**A**) The total number for colon resections was 87,853 in 2013 and 85,760 in 2018 and for sphincter-preserving rectal resections 31,195 in 2013 and 28,834 in 2018, decreasing slightly over the years. Data are absolute numbers per year. (**B**) The data show relative anastomotic leakage rates of 5.08% in 2013 and 6.74% in 2018 for colon resections and for sphincter-preserving rectal resections of 7.69% in 2013 and 9.15% in 2018. Data show relative rate per year. A linear trend towards higher leakage rates is shown. Chi-square test for trend, *p* ≤ 0.0001 = ****. (**C**, **D**) Anastomotic leakage rates with regard to secondary diagnosis, age range, and gender for colon resections (**C**) and sphincter-preserving rectal resections (**D**). Data are mean ± SD, dots are individual years. Bright blue and bright gray bar are mean leakage rates for all colon resections and all rectal resections. Two-sided Fisher’s exact test (secondary diagnosis, gender), chi-square test (age), *p* ≤ 0.0001 = ****. AL, anastomotic leakage

A total of 690,690 cases were registered by DESTATIS from 2013 to 2018 and included in this study, 513,951 cases with colon resections, and 176,739 with sphincter-preserving rectal resections. The total number of colon resections was 87,853 in 2013 and 85,760 in 2018 and sphincter-preserving rectal resections were performed 31,195 times in 2013 and 28,834 times in 2018, decreasing slightly over the years (Fig. 1A).

### Anastomotic leakage rates

An increase in reported AL rates for both types of surgery was seen in the first 3 years after the introduction of the ICD-code K91.83 for postoperative AL. Reported relative AL rates for the total number of colon resections were 5.1% in 2013 and 6.7% in 2018 and for rectal resections 7.7% in 2013 and 9.2% in 2018 (Fig. 1B). The mean AL rate (from 2013 to 2018) was 6.2% for colon resections and 8.8% for rectal resections (Table 1).

**Table 1:** Characteristics of study population and corresponding anastomotic leakage rates

		2013–2018						2013		2014		2015		2016		2017		2018	
		*n* (total)	*n* (AL)	Leakage rate	p*	OR**	95% CI	*n* (total)	*n* (AL)	*n* (total)	*n* (AL)	*n* (total)	*n* (AL)	*n* (total)	*n* (AL)	*n* (total)	*n* (AL)	*n* (total)	*n* (AL)
Total		690,690	47,453	6.87%				119,048	6860	116,936	7861	114,104	7943	113,244	8268	112,764	8105	114,594	8416
Type of surgery	Colon resection	513,951	31,934	6.21%				87,853	4460	86,976	5300	84,686	5335	84,197	5568	84,479	5494	85,760	5777
	Rectal resection	176,739	15,519	8.78%				31,195	2400	29,960	2561	29,418	2608	29,047	2700	28,285	2611	28,834	2639
Gender	Male	280,442	27,608	9.84%	*p* < *0.0001*	*1.43*	*1.40–1.46*	56,581	4007	55,352	4520	54,536	4555	NA	4785	53,974	4757	55,214	4984
	Female	243,599	16,497	6.77%				62,467	2853	NA	3341	59,568	3388	58,863	3483	NA	NA	59,360	3432
Age	21*–*40	34,084	1853	5.44%	*p* < *0.0001*			5403	267	5690	316	5610	295	5731	313	5711	342	5939	320
	41*–*60	183,452	12,486	6.81%				31,528	1768	31,044	2027	30,196	2124	30,239	2179	30,016	2146	30,429	2242
	61*–*80	357,221	25,947	7.26%				62,357	3757	61,252	4405	59,557	4371	58,347	4537	57,734	4347	57,974	4530
	> 80	109,603	6913	6.31%				18,799	1032	17,966	1072	17,666	1107	17,837	1206	18,197	1214	19,138	1282
Indication for surgery	***Colon resections***																		
	Colorectal cancer	189,183	11,874	6.28%	*p* = *0.1540*	*1.02*	*0.99–1.04*	31,655	1687	31,625	1900	31,041	1937	30,839	2106	31,620	2051	32,403	2193
	Crohn's disease	14,507	1002	6.91%	*p* = *0.0006*	*1.12*	*1.05–1.20*	2312	124	2500	177	2466	174	2478	168	2369	190	2382	169
	Diverticulosis	124,376	5766	4.64%	*p* < *0.0001*	*0.68*	*0.66–0.70*	22,866	822	21,895	1010	20,154	988	19,905	999	19,797	969	19,759	978
	***Rectal resections***																		
	Colorectal cancer	92,142	9260	10.01%	*p* < *0.0001*	*1.40*	*1.35–1.45*	16,067	1458	15,435	1503	15,207	1561	15,078	1610	14,884	1536	15,471	1592
	Crohn's disease	761	110	14.45%	*p* < *0.0001*	*1.76*	*1.44–2.16*	124	20	121	18	137	11	133	21	115	17	131	23
	Diverticulosis	34,773	2289	6.58%	*p* < *0.0001*	*0.69*	*0.65–0.72*	6610	338	6158	403	5659	399	5628	416	5355	384	5363	349
Secondary diagnosis	***Colon resections***																		
	Type II diabetes	84,542	5848	6.92%	*p* < *0.0001*	*1.15*	*1.12–1.18*	14,400	819	14,411	977	14,002	934	13,939	1053	13,730	1013	14,060	1052
	Obesity	50,724	3907	7.70%	*p* < *0.0001*	*1.30*	*1.25–1.34*	7883	461	8388	639	8380	665	8563	741	8576	654	8934	747
	Cachexia	7786	877	11.26%	*p* < *0.0001*	*1.94*	*1.81–2.09*	1158	96	1220	146	1348	151	1362	169	1363	159	1335	156
	Hypertension	247,447	16,113	6.51%	*p* < *0.0001*	*1.10*	*1.08–1.13*	41,300	2207	41,325	2578	40,393	2618	40,756	2899	41,299	2804	42,374	3007
	Chronic kidney disease	56,434	4489	7.95%	*p* < *0.0001*	*1.35*	*1.31–1.40*	9233	616	9438	752	9354	759	9369	778	9534	774	9506	810
	***Rectal resections***																		
	Type II diabetes	26,571	2731	10.28%	*p* < *0.0001*	*1.23*	*1.18–1.29*	4682	402	4512	446	4415	455	4312	475	4308	473	4342	480
	Obesity	16,308	1783	10.93%	*p* < *0.0001*	*1.31*	*1.24–1.38*	2696	231	2643	283	2670	308	2797	330	2699	297	2803	334
	Cachexia	2290	314	13.71%	*p* < *0.0001*	*1.66*	*1.48–1.88*	352	50	325	42	382	60	427	54	394	43	410	65
	Hypertension	83,621	7671	9.17%	*p* < *0.0001*	*1.10*	*1.06–1.13*	14,313	1118	14,059	1243	13,759	1302	13,853	1372	13,706	1301	13,931	1335
	Chronic kidney disease	15,309	1693	11.06%	*p* < *0.0001*	*1.33*	*1.26–1.40*	2636	281	2604	278	2555	272	2518	296	2511	291	2485	275

^*^Fisher’s exact test (Gender, age, underlying condition, secondary diagnosis); Chi-square test (age)

^**^Odds ratio. Confidence interval (CI) computed by Woolf logit

*AL* anastomotic leakage, *NA* data not available due to data protection (*n* ≤ 2) caused by cases with unknown gender

### Other postoperative complications

Postoperative abscess/surgical site infection rates following colon resections were 8.8% in 2013 and 7.7% in 2018 and for rectal resections 7.7% in 2013 and 6.6% in 2018. Wound dehiscence occurred with a rate of 6.5% in 2013 and 7.0% in 2018 for colon resections and with a rate of 5.6% in 2013 and 5.6% in 2018 for rectal resections. The rate of postoperative fistula formation was 5.6% in 2013 and 5.2% in 2018 for colon resections and 5.1% in 2013 and 4.6% in 2018 for rectal resections (Fig. S1).

### Indication for surgery

Concerning the primary indication (main diagnosis) for colon and rectal resections, relevant differences in AL rates could be detected. For colon resections, patients with diverticulosis showed a significantly lower than average leakage rate (4.6%, OR 0.68). For patients with Crohn’s disease, the leakage rate was above average for colon resections (6.9%, OR 1.12). Patients with colorectal cancer showed a leakage rate of 6.3% which was not significantly different from the average leakage rate for colon resections of 6.2% (OR 1.02). For rectal resections, patients with diverticulosis showed a significantly lower than average leakage rate (6.6%, OR 0.69). For patients with Crohn’s disease (14.5%, OR 1.76) and patients with colorectal cancer (10.1%, OR 1.40), the leakage rate was higher than average for rectal resections (Table 1).

### Risk factors for anastomotic leakage

Regarding the individual risk factors for AL, patients with type 2 diabetes mellitus, obesity, cachexia, hypertension, and chronic kidney disease had significantly higher AL rates compared to cases without these secondary diagnoses (Fig. 1C, D). Leakage rates for patients with type 2 diabetes mellitus were 6.9% (colon)/10.3% (rectum), obesity 7.7%/10.9%, cachexia 11.3%/13.7%, hypertension 6.5%/9.2%, and chronic kidney disease 8.0%/11.1% (Table 1). Additionally, a significant correlation between patient age and AL could be shown (*p* < 0.0001). Leakage rates were highest for patients between 61 and 80 years of age (Fig. 1C, D, Table 1). Regarding gender, male patients had significantly higher leakage rates than female patients for both colon resections (male: 7.4%, female 5.2%, *p* < 0.0001) and rectal resections (male: 11.3%, female: 6.4%, *p* < 0.0001) (Fig. 1C, D). The general comorbidity was higher in patients with anastomotic leakage as evaluated with the Strausberg and weighed Elixhauser Comorbidity Scores (Table S2).

### Management of anastomotic leakage

**Fig. 2:**
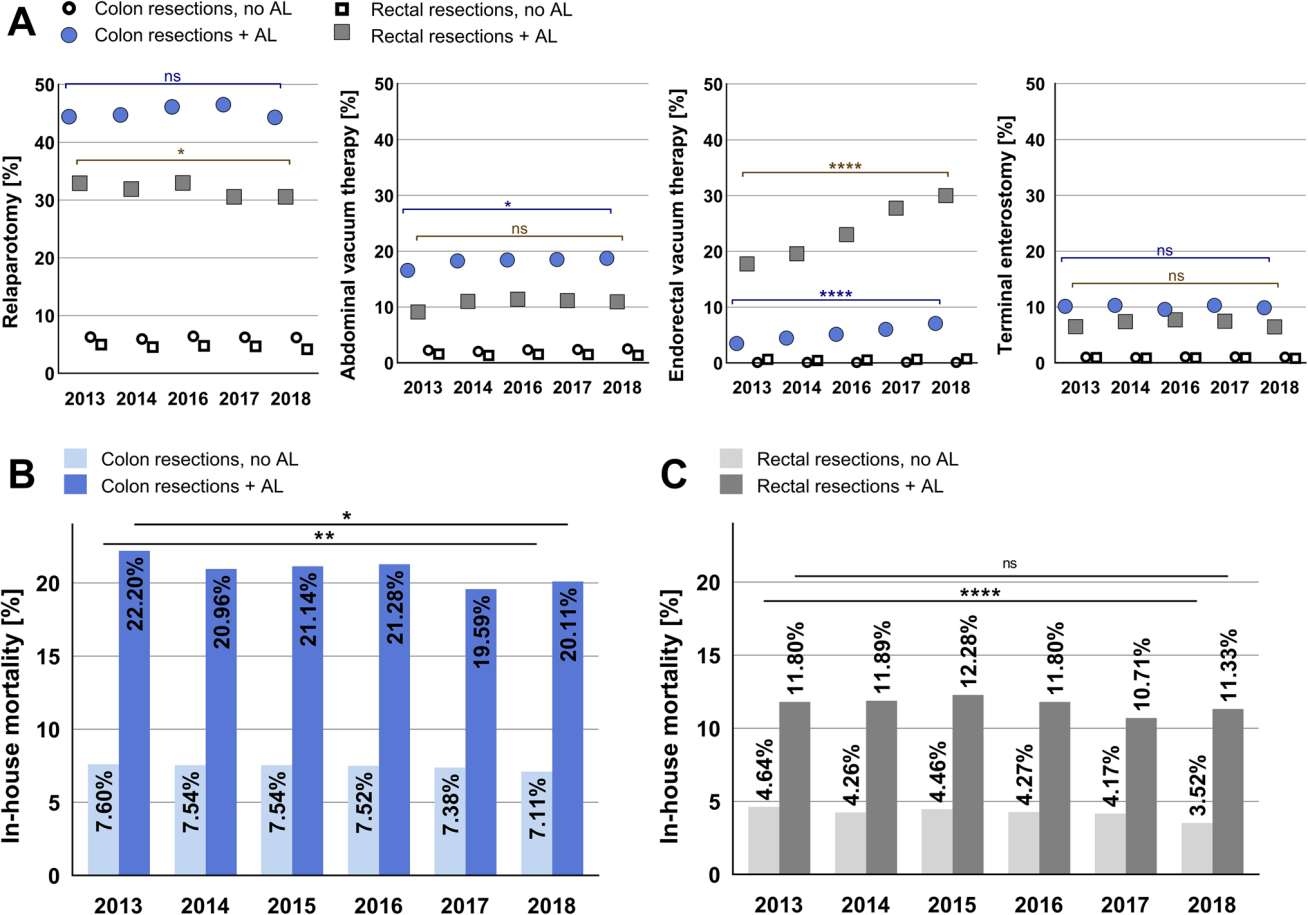
Management of anastomotic leakage and in-house mortality. (**A**) Procedures following anastomotic leakage after colon and rectal resections (relaparotomy, abdominal vacuum therapy, endorectal vacuum therapy, terminal enterostomy). Rates of procedures in cases with no anastomotic leakage (AL) are depicted for comparison. Data show relative rate per year. Chi-square test for trend. *p* < 0.05 = *, *p* ≤ 0.0001 = ****. Data for 2015 not available. (**B**, **C**) In-house mortality in % of cases undergoing colon resections (**B**) or rectal resections (**C**) without and with anastomotic leakage (AL). Data show relative rate per year. Chi-square test for trend. *p* < 0.05 = *, *p* ≤ 0.01 = **, *p* ≤ 0.0001 = ****. AL, anastomotic leakage

44.4% of patients with AL after colon resections and 32.9% of patients with AL after rectal resections underwent relaparotomy (2013). Relaparotomy rates for cases with AL only decreased for rectal resections over time (Fig. 2A). Abdominal vacuum therapy was performed in 16.6% of cases with AL after colon resections in 2013. For cases with AL after rectal resection, abdominal vacuum therapy was performed in 9.1% in 2013 (Fig. 2A). Regarding endorectal vacuum therapy, a significant increase over time could be detected. In 2013, endorectal vacuum therapy was performed in 3.5% of cases with colon resections and 17.8% of cases with rectal resections and postoperative AL. In 2018, endorectal vacuum therapy was performed in 7.1% of cases with colon resections and 30.0% of cases with rectal resections and AL (Fig. 2A). Terminal enterostomy was performed in 10.2% of cases after colon resections and AL and 6.4% of cases after rectal resections and AL in 2013. Rates for terminal enterostomy did not change significantly over time (Fig. 2A).

### Mortality

The in-house mortality for patients undergoing colon resections without AL was 7.6% in 2013 and 7.1% in 2018. Mortality for patients with AL after colon resections was 22.2% in 2013 and 20.1% in 2018. A slight negative trend in mortality rates could be detected (Fig. 2B). The in-house mortality for patients undergoing rectal resections without AL was 4.6% in 2013 and 3.5% in 2018. Mortality for patients with AL after rectal resections was 11.8% in 2013 and 11.3% in 2018. Here, only a negative trend in mortality rates could be detected in patients with rectal resections without AL (Fig. 2C).

### Length of hospital stay and hospital reimbursement

**Fig. 3:**
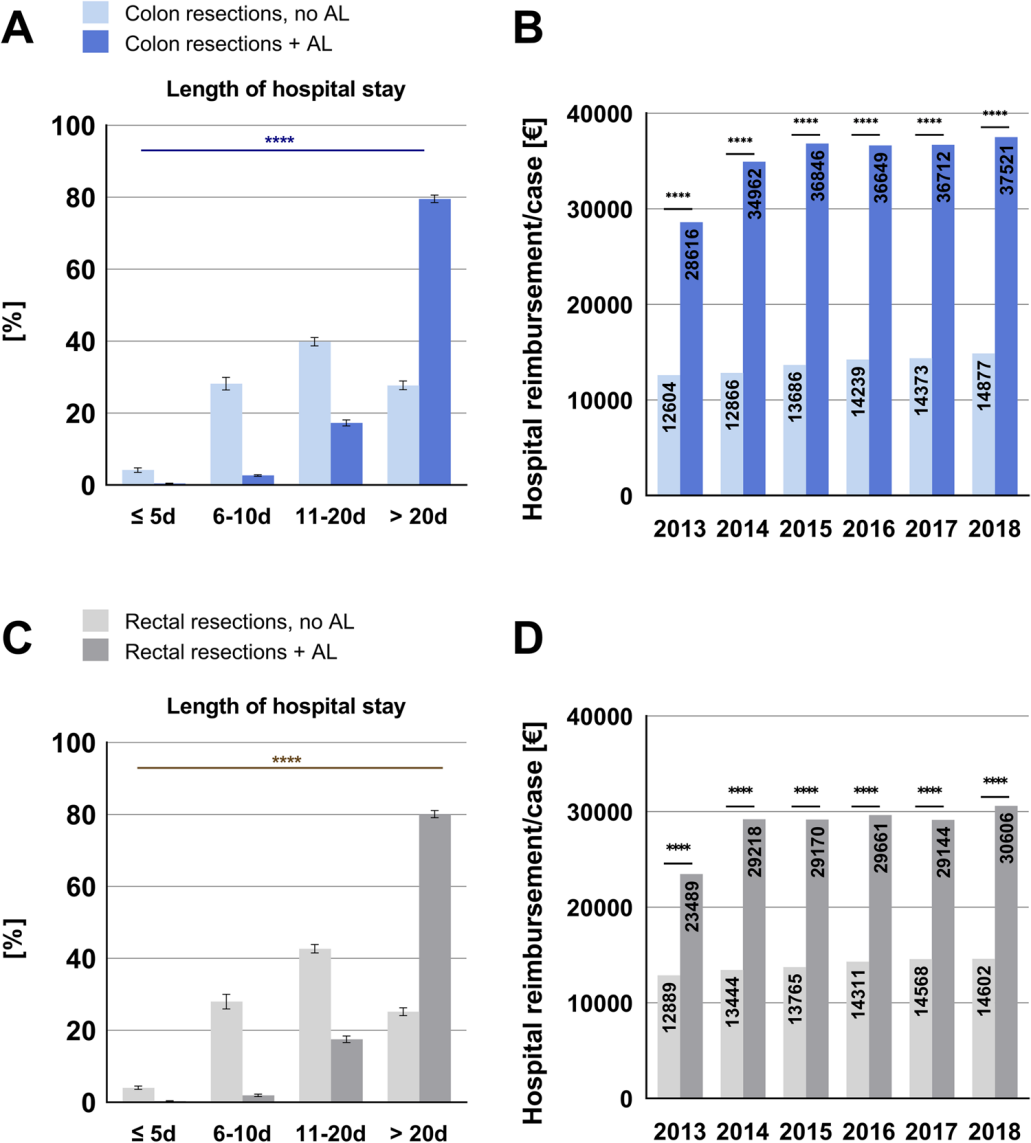
Length of hospital stay and hospital reimbursement. (**A**, **C**) Distribution of cases to the length of hospital stay (≤ 5 days, 6–10 days, 11–20 days, ≥ 20 days). Data is depicted as percentage of total cases for colon resection ± anastomotic leakage (**A**) and rectal resection ± anastomotic leakage (**C**). A significant association between anastomotic leakage and length of hospital stay can be shown. Data are mean ± SD. Chi-square test. *p* ≤ 0.0001 = ****. (**B**, **D**) Mean hospital reimbursement sum per case for colon and rectal resections with and without anastomotic leakage. Data are mean reimbursement sum per year, *t*-test, *p* ≤ 0.0001 = ****. AL, anastomotic leakage

The occurrence of AL had a significant influence on the length of hospital stay in both colon and rectal resections. In 80% of cases with colon resections and AL, the length of hospital exceeded 20 days while in cases without AL, 28% of patients stayed in the hospital for more than 20 days, most likely due to other complications. In 80% of cases with rectal resections and AL, the length of hospital stay was longer than 20 days while in cases without AL, 25% of patients stayed in the hospital for more than 20 days (Fig. 3A, C).

The mean hospital reimbursement sum for colon resections was 12,603€ without AL and 28,616€ with AL in 2013 and 14,876€ without versus 37,521€ with AL in 2018, showing an increase in the mean hospital reimbursement sum over time. Regarding rectal resections, the mean hospital reimbursement was 12,889€ without and 23,488€ with AL in 2013 and 14,602€ versus 30,606€ in 2018 (Fig. 3B, D; Table 2). To estimate the potential saving that could be achieved if AL could be prevented in all cases, we calculated a hypothetical sum from the mean hospital reimbursement rates of patients with and without AL (Table 2).

**Table 2:** Hospital reimbursement and hypothetical savings

Year	Anastomotic leakage (AL)	n	[%]	Mean hospital reimbursement sum [€]	p*	Hypothetical savings if no AL** [€]
Partial colon resection						
2013	No	83,290	94.92%	12,603.67	< 0.0001	71,384,393
	Yes	4458	5.08%	28,616.32		
2014	No	81,582	93.91%	12,865.77	< 0.0001	116,974,953
	Yes	5294	6.09%	34,961.53		
2015	No	79,274	93.70%	13,686.06	< 0.0001	123,464,733
	Yes	5331	6.30%	36,845.83		
2016	No	78,559	93.39%	14,238.63	< 0.0001	124,691,521
	Yes	5564	6.61%	36,649.04		
2017	No	78,901	93.49%	14,372.71	< 0.0001	122,644,898
	Yes	549	6.51%	36,712.40		
2018	No	79,856	93.26%	14,876.75	< 0.0001	130,705,439
	Yes	5772	6.74%	37,521.49		
Rectal resection						
2013	No	28,767	92.30	12,889.10	< 0.0001	25,428,224
	Yes	2399	7.70%	23,488.61		
2014	No	27,383	91.45%	13,444.44	< 0.0001	40,396,266
	Yes	2561	8.55%	29,218.07		
2015	No	26,794	91.13%	13,764.78	< 0.0001	40,161,747
	Yes	2607	8.87%	29,170.13		
2016	No	26,317	90.70%	14,311.33	< 0.0001	41,427,679
	Yes	2699	9.30%	29,660.60		
2017	No	25,648	90.76%	14,568.00	< 0.0001	38,057,909
	Yes	2611	9.24%	29,143.99		
2018	No	26,169	90.84%	14,602.00	< 0.0001	42,235,717
	Yes	2639	9.16%	30,606.44		

^*^Unpaired *t*-test

^**^Hypothetical saving in case of no anastomotic leakage = *n* (“AL YES”) × (mean hospital reimbursement sum “AL YES” − mean hospital reimbursement sum “AL NO”)

## Discussion

With more than 690,000 inpatient cases undergoing colon resections and sphincter-preserving rectal resections, our study is currently the largest nation-wide population-based study to analyze AL rates after surgery of the lower gastrointestinal tract. Despite increasing research in the field of anastomotic healing and improvement of surgical techniques, our data show no decrease in leakage rates from 2013 to 2018 with a mean AL rate for colon resections of 6.2% and rectal resections of 8.8%.

Regarding individual risk factors for AL, known risk factors such as male gender, diabetes, hypertension, obesity, and chronic kidney disease could be confirmed by our data [4, 7, 8]. Interestingly, cachexia showed the highest odds ratio for AL of 1.94 in cases with colon resections and 1.66 in cases with rectal resections (Table 1). When looking at the management of AL, there is a significant increase in endoscopic therapy in terms of endoluminal vacuum therapy over the years leading to a rate of 30% for AL after rectal resections in 2018; however, relaparotomy rates only slightly decreased in the studied period (Fig. 2A). Two potential factors could explain this phenomenon. Firstly, for an effective endoluminal vacuum therapy, the creation of a diverting enterostomy might be necessary thus requiring relaparotomy. Secondly, relaparotomy for peritoneal lavage might be required for patients with AL before or in combination with endorectal vacuum therapy thus not leading to a significant reduction in relaparotomy rates. Hence, our data suggests that although endoluminal vacuum therapy for AL after colorectal surgery is increasingly applied, it does not prevent revision surgery for lavage and creation of a protective enterostomy in all cases.

The AL rates that were coded increased over the observation period from 2013 to 2018, reaching a relatively stable level by 2015. The most probable cause is underreporting in the first years after the introduction of the ICD code K91.83 in 2013. The bias of under-reporting of AL in the following years is unlikely, as the hospitals would have deliberately waived a higher DRG-based reimbursement sum when treating for AL but not coding it in the case data. Over-reporting of diagnoses and procedures on the other hand is strictly controlled by the medical service of the health insurance funds in Germany but could still lead to a bias in our study. Other studies have described similar leakage rates but to our knowledge, no study had nearly as many cases or patients included in their data sets. Bonström et al. describe AL in 10% of the included 6948 patients undergoing low anterior resection in a population-based study from 2019 [20]. Gessler et al. describe AL rates of 7.0% for right hemicolectomy, 7.4% for left hemicolectomy, and 18.8% for rectal resection in a patient collective of 600 patients [2]. In a nationwide analysis from the USA, Midura et al. however show a much lower overall leakage rate of 3.8% [7]. The heterogeneity of assumed leakage rates has been reported several times recently [4, 21]. One confounder in most studies on AL rates is that postoperative diagnostic regimens are not standardized leading to under-diagnosis, especially of grade A leakage (according to the International Study Group of Rectal Cancer 2010) which is defined by not affecting the postoperative management [22]. However, with our study, we could show that despite increasing knowledge on the risk factors for AL, there was no trend towards decreasing leakage rates in the studied time period.

Regarding the economic burden of AL, only the DRG-based hospital reimbursement volume is accessible by our type of data query. A significant increase in the hospital reimbursement sum for cases with AL compared to cases without AL can be seen. We have calculated potential savings that could be achieved if no AL would occur (130,705,439 € for colon resections and 42,235,717 € for rectal resections in 2018, Table 2). However, the real costs of AL for the individual hospital cannot be derived from the DRG data. It has been described that the real cost of AL for the individual hospital is significantly higher and is not covered by the DRG-based reimbursement system. La Regina et al. could demonstrate in a study including 95 patients undergoing colorectal cancer surgery, that the mean profit from the DRG-based reimbursement was 542€ per case without postoperative complications and the mean loss for cases with AL was 12,181€ per case for the hospital treating patients that developed AL [23]. In a study from England, Ashraf et al. could also demonstrate inadequate hospital reimbursement for cases with AL after low anterior rectum resections [24]. The slight increase in the overall hospital reimbursement sum is most likely due to the fact that the hospital reimbursement is calculated based on a base rate per inpatient hospital case, which increases steadily over time.

Ultimately, the question remains as to why AL rates have stagnated at such a high level. A Dutch study from 2022 investigated the impact of perioperative potentially modifiable risk factors on AL after colorectal surgery during a study period from January 2016 to December 2018 [25]. They identified modifiable risk factors such as low preoperative hemoglobin, surgical site contamination, hyperglycemia, and inadequate timing of perioperative antibiotic prophylaxis. Interestingly, most of these factors were already known to increase the risk of AL, but their prevention was still not applied before and during surgery. Although we could not draw these data from the DESTATIS dataset in our study, we suspect that the Dutch data are transferable to the situation in Germany. We therefore hypothesize that despite known preventive measures to reduce AL rates, adherence is still lacking in Germany, which could at least partly explain the stagnant AL rates in our study. Furthermore, we hypothesize that even if all standards to prevent AL are met, there is a residual risk for AL that has not yet been identified. Moreover, some patient-specific risk factors cannot be modified before surgery. To date, there are no established local or systemic pharmacological therapies to prevent anastomotic complications and improve the postoperative healing process in patients undergoing colorectal surgery. Therefore, with our study, we aim to raise awareness that AL is an unresolved problem in Germany, which represents an unchanged burden for patients as well as for health care providers and insurance companies.

## Conclusions

This study presents a large population-based data set on AL rates following lower gastrointestinal surgery and gives a timely overview of the current data on AL rates and associated socioeconomic costs of AL after lower gastrointestinal surgery in Germany. The data show a great need for further research in the field of AL and for better adherence to perioperative standards to minimize known risks to efficiently reduce leakage rates and thus improve patient outcomes in the future. Furthermore, treatment for AL and care for affected patients must improve to reduce the high in-house mortality associated with AL.

### Supplementary Information

Below is the link to the electronic supplementary material.

Supplementary file1 (DOCX 314 KB)

## Data Availability

Due to the regulations of the Federal Statistical Office of Germany (DESTATIS), original data from the data query cannot be made available. However, the SAS code that was used to retrieve the data can be requested from the authors via email.
