# Supplementary material for: A nationwide population-based study on the clinical and economic burden of anastomotic leakage in colorectal surgery
Source: Langenbecks Arch Surg. 2023 Jan 23;408(1):55. doi: 10.1007/s00423-023-02809-4 (PMC9868041; doi:10.1007/s00423-023-02809-4)
Supplement: Supplementary file 1 — Supplementary file1 (DOCX 314 KB) [file 423_2023_2809_MOESM1_ESM.docx]

## A nationwide population-based study on the clinical and economic burden of anastomotic leakage in colorectal surgery

Marie-Christin Weber^1^, Maximilian Berlet^1^, Christian Stoess^1^, Stefan Reischl^1, 2^, Dirk Wilhelm^1^, Helmut Friess^1^, Philipp-Alexander Neumann^1^

^1^ Technical University of Munich, Germany, TUM School of Medicine, Klinikum rechts der Isar, Department of Surgery

^2^ Technical University of Munich, Germany, TUM School of Medicine, Klinikum rechts der Isar, Department of Diagnostic and Interventional Radiology

**– Supplementary Information –**

### SUPPLEMENT

*Table S1: OPS- and ICD-Codes*

| OPS*-/ICD**-Code | Description |
| --- | --- |
| *Type of surgery* | |
| OPS 5-455 | partial colon resection |
| OPS 5-484 | sphincter preserving rectal resection |
| *Postoperative complications* | |
| ICD K91.83 | anastomotic leakage (postoperative) |
| ICD T81.3 | wound dehiscence (postoperative) |
| ICD T81.4 | abscess, infection (postoperative) |
| ICD T81.8 | fistula (postoperative) |
| *Management* | |
| OPS 5-541.2 | relaparotomy |
| OPS 5-916.a3 | abdominal vacuum therapy |
| OPS 5-916.a4 | endorectal vacuum therapy |
| OPS 5-461 | terminal enterostomy |
| *Indication for surgery* | |
| ICD C18.-, C19, C20 | colorectal cancer |
| ICD K50.- | crohn's disease |
| ICD K57.- | diverticulosis |
| *Secondary diagnosis* | |
| ICD E11.- | type II diabetes |
| ICD E66.- | obesity |
| ICD R64 | cachexia |
| ICD I10.- | hypertension |
| ICD N18.- | chronic kidney disease |

** OPS = Operation and Procedure Classification System in Germany*

*** ICD = International Statistical Classification of Diseases and Related Health Problems, 10th revision, German Modification (ICD-10-GM)*

*Table S2: Comorbidity Scores in relation to anastomotic leakage*

|  | Anastomotic leakage | 2013  *mean (SD)* | 2014  *mean (SD)* | 2015  *mean (SD)* | 2016  *mean (SD)* | 2017  *mean (SD)* | 2018  *mean (SD)* |
| --- | --- | --- | --- | --- | --- | --- | --- |
| Elixhauser Comorbidity Index (weighted) | | | | | | | |
| Colon resection | No | 7.86 (8.87) | 8.07 (9.09) | 8.35 (9.25) | 8.57 (9.40) | 8.47 (9.35) | 8.43 (9.36) |
|  | Yes | 11.88 (9.55) | 12.60 (10.03) | 12.83 (10.11) | 13.13 (10.22) | 13.15 (10.25) | 12.90 (10.24) |
|  |  | *p* < 0.0001 | *p* < 0.0001 | *p* < 0.0001 | *p* < 0.0001 | *p* < 0.0001 | *p* < 0.0001 |
| Rectal resection | No | 7.47 (8.58) | 7.72 (8.77) | 8.02 (8.91) | 8.05 (8.98) | 8.14 (8.99) | 7.99 (8.88) |
|  | Yes | 10.84 (9.43) | 11.36 (9.67) | 11.65 (9.86) | 12.06 (9.94) | 12.26 (10.07) | 12.19 (9.96) |
|  |  | *p* < 0.0001 | *p* < 0.0001 | *p* < 0.0001 | *p* < 0.0001 | *p* < 0.0001 | *p* < 0.0001 |
| Strausberg Comorbidity Score | | | | | | | |
| Colon resection | No | 9.85 (19.36) | 9.94 (19.66) | 10.68 (20.29) | 11.16 (20.54) | 11.23 (20.62) | 11.23 (20.63) |
|  | Yes | 29.93 (23.34) | 31.04 (24.12) | 32.18 (24.95) | 33.01 (24.82) | 32.72 (25.09) | 32.96 (24.8) |
|  |  | *p* < 0.0001 | *p* < 0.0001 | *p* < 0.0001 | *p* < 0.0001 | *p* < 0.0001 | *p* < 0.0001 |
| Rectal resection | No | 8.12 (18.26) | 8.12 (18.27) | 8.92 (18.96) | 8.94 (18.9) | 9.17 (19.1) | 9.06 (18.9) |
|  | Yes | 23.92 (21.88) | 25.00 (22.86) | 25.07 (23.94) | 26.26 (23.76) | 27.53 (23.94) | 27.28 (24.07) |
|  |  | *p* < 0.0001 | *p* < 0.0001 | *p* < 0.0001 | *p* < 0.0001 | *p* < 0.0001 | *p* < 0.0001 |


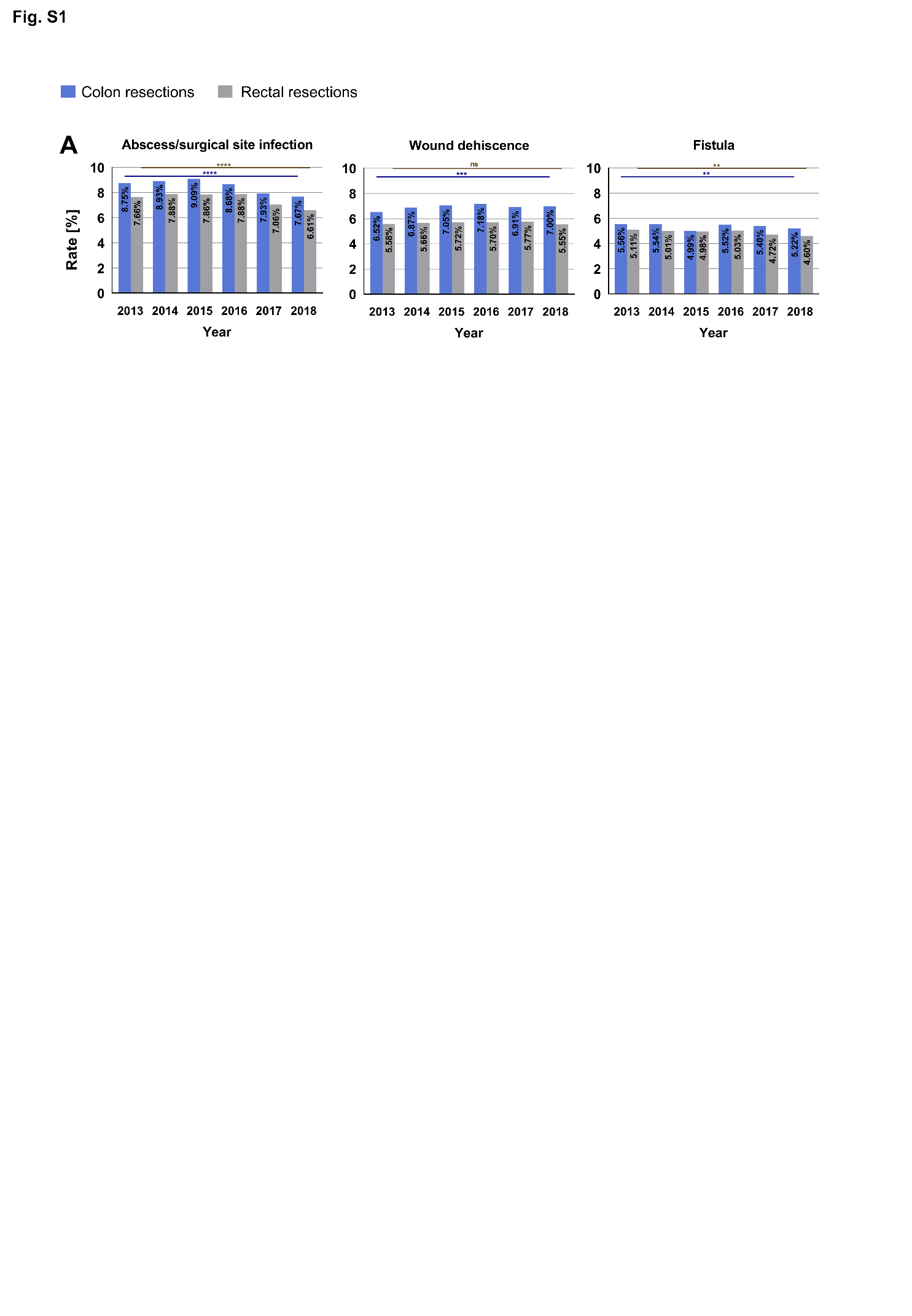


**Fig. S1: Additional postoperative complications.** (A) Relative rates of other postoperative complications: abscess/surgical side infection, wound dehiscence and fistula after colon resections and sphincter-preserving rectal resections. Data are relative rate per year. Chi-square test for trend, *p* ≤ 0.01 = **, *p* ≤ 0.001 = ***. *p* ≤ 0.0001 = ****.
